# The Curious Road from Basic Pathogen Research to Clinical Translation

**DOI:** 10.1371/journal.ppat.1004997

**Published:** 2015-06-25

**Authors:** Grant McFadden

**Affiliations:** Department of Molecular Genetics and Molecular Biology, University of Florida, Gainesville, Florida, United States of America

**Image 1:**
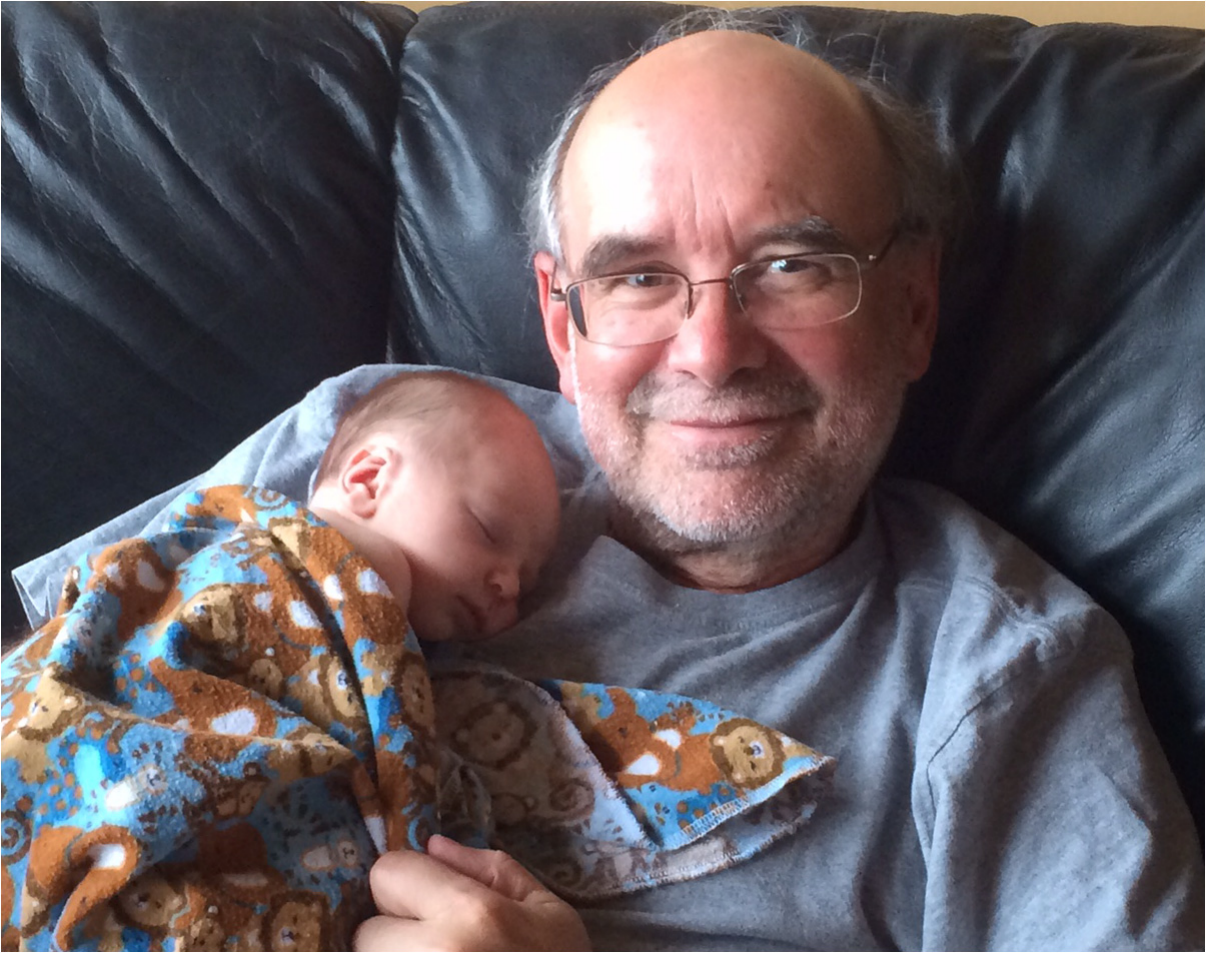
Grant McFadden and his grandson.

I was very lucky to have stumbled into curiosity-driven scientific research for a career, because I suspect that I have always had a poorly developed herd instinct. In my case, when I set up my first research lab as a new primary investigator in 1980, I elected to study a family of viruses that infect only rabbits. Why rabbits, you ask? I had become fascinated by events that had happened a half-century earlier, when a rabbit poxvirus called myxoma virus had been deliberately released into the Australian outback in a failed attempt to wipe out the hoards of feral rabbits that had proliferated out of control and devastated the ecological balance of the countryside. This release failed to reduce rabbit populations as planned but it did prove another point that later turned out to be significant: myxoma virus was completely harmless to all other vertebrates, including humans. So, it was pretty much guaranteed that the research in my lab would never be relevant to any human disease. But fortunately for me, I received research funding to investigate how this virus causes disease only in this one particular host species.

After several years of investigating the molecular features of this virus, we discovered that the virus genome encoded a large family of proteins with remarkable similarity to elements of the host immune system. In due course, the field began to appreciate that many viruses capable of selectively infecting vertebrates have co-evolved to counteract or evade the immune defenses of their hosts. Brett Finlay and I coined the term “anti-immunology” to describe an amazingly diverse family of pathogen-derived proteins that evolved to control the vertebrate immune system. Over time, my lab began genetically deleting viral genes from myxoma virus one at a time, in order to observe the effects of the various targeted gene knockouts on the virus, but then two unexpected discoveries began to drag my career more into the world of translational research.

The first discovery, made with Alexandra Lucas, revealed that some anti-host viral proteins were powerful inhibitors of the host immune responses to the virus. These viral proteins could be purified individually and used as drugs to inhibit the same immune cells when they become hyper-activated during inflammatory diseases like atherosclerosis. This led to the formation of a new startup biotech company (Viron Therapeutics), and introduced me into the world of applied translational research and clinical trials. Although it is still not a given that viral proteins will end up being approved and licensed for clinical use, I am now an advocate of virus-derived proteins as a new source of drugs to treat immune-based diseases that have no known connection with viruses.

The second discovery, beginning about a decade ago, occurred when we discovered that myxoma virus also grows in many classes of human cancer cells, but not in normal tissues (unless you are a rabbit). In fact, when my longtime collaborator Peter Forsyth injected it into human gliomas transplanted into the brains of test immunodeficient mice, the virus grew selectively within the transplanted human brain tumors just like it does in the internal tissues of myxoma-infected rabbits! Since it is totally nonpathogenic for humans, this discovery has convinced us to develop myxoma virus as a new potential therapeutic for a variety of human cancers. I have now formed a partnership with a biotech company that specializes in virotherapy for cancer (DNAtrix), and our first clinical trial goal will be to improve the outcomes for cancer patients receiving bone marrow transplants. I am very excited about this new approach against cancer in general, but only time will tell whether using live oncolytic viruses to treat cancer will become a licensed clinical tool for oncologists in the future.

I think my own take-home message is that the results of true fundamental research still remain virtually impossible to predict, despite what pundits or politicians might have you believe. In fact, I continue to passionately support curiosity-based research for its own sake of expanding the boundaries of knowledge, not just to fulfill a pre-approved agenda. To me, the single most important justification for fundamental research in biology remains this: Mother Nature is mysterious and magnificent but some of Her secrets can still be revealed if we only allow curious minds to ask the right questions. But with the growing din of anti-science sentiments, those of us who have been lucky enough to pursue fundamental research as a career now more than ever need to speak up. If we want the next generation of scientists to lead the way to the transformative discoveries of the future, we all need to articulate more clearly to nonscientists why, in our modern world, basic research matters more than ever.

